# Comparison of stationary digital chest tomosynthesis to portable chest radiographs: A patient study

**DOI:** 10.1371/journal.pone.0344178

**Published:** 2026-04-02

**Authors:** Courtney Wing, Elias Taylor Gunnell, Alex Billingsley, Umer Ahmed, Christina Inscoe, Muthu Sakthivel, Bryan Hoag, Ertan Pamuklar, Thad Benefield, Jianping Lu, Xiaohui Wang, Otto Zhou, Yueh Z. Lee

**Affiliations:** 1 Department of Radiology, The University of North Carolina at Chapel Hill, Chapel Hill, North Carolina, United States of America; 2 Department of Radiology, The Medical University of South Carolina, Charleston, South Carolina, United States of America; 3 Joint Department of Biomedical Engineering, The University of North Carolina at Chapel Hill, Chapel Hill, North Carolina, United States of America; 4 Department of Radiology, Boston Medical Center, Boston, Massachusetts, United States of America; 5 Department of Physics and Astronomy, The University of North Carolina at Chapel Hill, Chapel Hill, North Carolina, United States of America; 6 Charlotte Radiology, Charlotte, North Carolina, United States of America; 7 Carestream Health Inc., Rochester, New York, United States of America; University of Pittsburgh Medical Center Pinnacle Health Medical Services, UNITED STATES OF AMERICA

## Abstract

**Background:**

Portable chest radiographs are a vital tool for clinical evaluation. However, their value remains limited due to overlapping anatomical structures obscuring underlying pathologies. Chest tomosynthesis has been proposed as an alternative to CT for high resolution chest imaging; however, a portable tomosynthesis unit is currently lacking in the standard of care. Utilizing a carbon nanotube linear x-ray source array, our team has developed a stationary digital chest tomosynthesis (s-DCT) unit that enables tomosynthesis imaging without need for a moving x-ray source during image acquisition.

**Purpose:**

The goal of our study was to evaluate the performance of portable s-DCT in patients with concurrent portable radiographs and recent cross-sectional chest CT imaging.

**Methods:**

This study recruited twenty-seven outpatients with recent chest CT images. Twenty-two patients were imaged with a conventional radiograph unit and the s-DCT system. Three board-certified thoracic radiologists rated their confidence in evaluating ten criteria: nine anatomic criteria and one on indwelling hardware. Readers were asked if information was gained from chest tomosynthesis, and if a follow-up CT was recommended. Random effects modeling was performed to assess factors contributing to reader confidence.

**Results:**

Twenty-two adult patients were successfully imaged. Overall, reader confidence with s-DCT was significantly higher than CXR (p = < 0.001). Higher confidence in tomosynthesis was associated with perception that tomosynthesis gave additional information over CXR (p = 0.05). There was no significant trend toward not recommending a CT (p = 0.47).

**Conclusion:**

Stationary digital chest tomosynthesis is a superior alternative to portable chest x-ray for patients that cannot undergo CT examination.

## Introduction

Physicians providing acute care in intensive care units rely on accurate and accessible chest imaging to direct treatment decisions. Complications such as a developing infection, pleural effusion, pneumothorax, pericardial effusion, or misplacement of an indwelling line or chest tube are not always clinically apparent, therefore repeat imaging has become an essential part of ICU standard of care [1]. Portable chest x-ray (P-CXR) remains by far the most widely used imaging modality in the management of critically ill patients [2]. However, P-CXR is also notoriously limited by low contrast resolution, lack of 3D detail, and low diagnostic sensitivity and specificity [3–5]. Computed tomography provides better discrimination and 3-D detail for thoracic pathology, however many patients in the ICU are often too medically unstable for transport to the radiology department for CT imaging [6,7].

Conventional digital chest tomosynthesis (DCT) is an imaging technique that collects a series of projection images of the chest by mechanically moving a single X-ray source over a limited angular span. A typical DCT system acquires roughly 60 projections over 30–45 degrees in approximately 10 seconds [8]. This provides the radiologist with a quasi-3-D image of the chest while exposing the patient to less than 10% of the radiation of a conventional CT [9,10]. Digital tomosynthesis has demonstrated promising clinical applications in a variety of fields. Breast tomosynthesis is poised to become the standard of care in mammography, especially in patients with higher risk or dense breasts [11,12]. Digital tomosynthesis has also shown promise in other fields and applications including: the field of orthopedics, cardiovascular imaging, abdominal imaging, and chest imaging in the evaluation of cystic fibrosis, pulmonary nodules, airway lesions and asbestos related pleural plaques [3,13–20].

We have developed a carbon nanotube (CNT) based linear x-ray source array that enables stationary digital chest tomosynthesis (s-DCT) from a portable imaging unit [16,18,21,22]. We retrofitted a repurposed source array to a conventional portable digital radiography machine, with a flat panel detector placed under the patient. Our imaging protocol uses an entrance dose of 0.6 mGy, which is comparable to the entrance dose of standard chest x-ray systems (0.31 to 0.88mGy) and conventional chest tomosynthesis systems (0.31 to 1.27mGy) [23,24]. The primary goal of our study was to compare our stationary digital chest tomosynthesis system to portable CXR in the evaluation of patients with a variety of lung pathologies. Secondary goals focused on whether s-DCT gave readers additional information that CXR did not, and whether a follow up Chest CT was recommended for further evaluation after tomosynthesis images were reviewed.

## Materials and methods

This prospective multireader multicase (MRMC) study was approved by the Institutional Review Board. Informed, written consent was obtained from each patient before enrollment.

### Subjects

Adult patients undergoing clinically indicated outpatient non-contrast chest CT for any pathological condition of the lung between July 17 2014 and June 22, 2015 were recruited to have a portable CXR using a conventional device and a s-DCT scan with our CNT x-ray source array. Patients underwent all three scans within a two-week period.

Participants were randomly selected from non-contrast chest CT clinical schedules in order to identify patients with a wide variety of clinical pathologies. Patients were selected based on the following inclusion and exclusion criteria. Patients were required to be over 18 years of age and had a clinically indicated non-contrast chest CT scheduled within a time frame that allowed for follow-up imaging with s-DCT within 2 weeks. Female patients of childbearing age had a negative urine pregnancy test within 1 week of their s-DCT scan. Exclusion criteria included patients who were unable to provide consent, pregnant or lactating women, patients with a BMI greater than 35, or being unable to fit on the 35x43cm s-DCT detector, and patients with planned procedures or surgeries between scans.

### Imaging protocol

**Fig 1:**
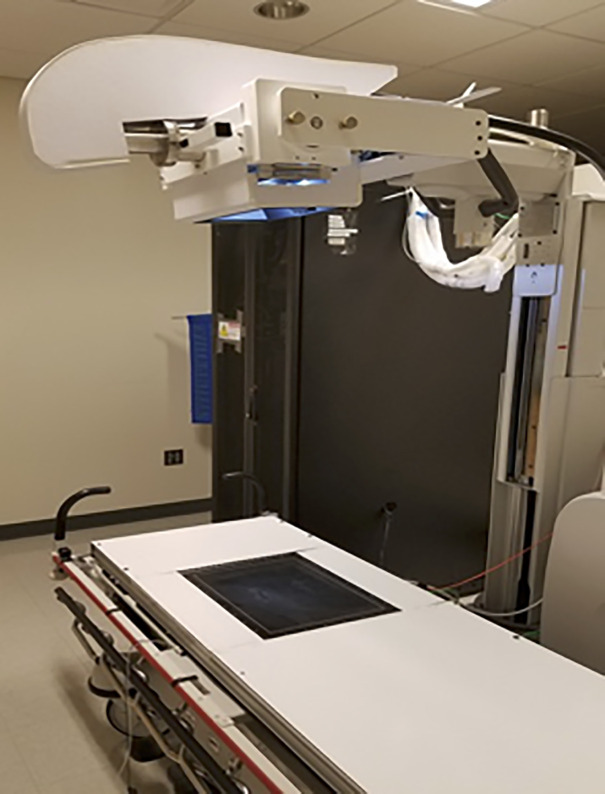
Stationary Digital Chest tomosynthesis system, CNT array retrofitted into a portable X-ray system.

Patients underwent s-DCT using our CNT x-ray source array and portable CXR. The prototype s-DCT system consists of 1) a vacuum tube with a length of 29 cm consisting of 29 equally spaced CNT x-ray emitters in a linear array arrangement operating at 80 kVp, and 2) a Carestream DRX Plus 3543C flat-panel digital detector (Carestream Health Inc, Rochester, NY) (Fig 1). The 29-emitter source array covers a 12-degree angular span and is positioned with a source to detector distance of 130 cm. The x-ray tube anode consists of a tungsten target and the total beam filtration is 2.5 mm aluminum equivalent. The measured focal spot sizes of the 29 emitters averaged 2.5 mm by 0.5 mm. The flat panel x-ray detector is 35 cm by 43 cm in effective imaging area with a 139 µm pixel pitch and acquisition speed of 5 fps. The system resolution was measured to be 1.7 cycles/mm in the scan direction and 3.4 cycles/mm in the perpendicular direction. Patients were placed in the supine position and imaging was acquired in an anterior to posterior (AP) orientation, with the x-ray source positioned above the patient and the detector. During imaging patients were instructed to hold their breath for six seconds during which time x-ray pulses were fired sequentially. A total of 29 projection images were acquired per patient. Similarly, the chest x-ray was performed on a Carestream DRX Mobile X-ray System (Carestream Health Inc, Rochester, NY) during the same visit, also acquired in an AP orientation.

All s-DCT projection images were moved to an off-line computer and were reconstructed using Matlab (Mathworks, Inc. Natick MA) custom-adapted fan volume reconstruction (AFVR) code [25]. The resulting reconstructions produced a coronal slice width of 2 mm and generated a tomosynthesis image stack of 90 coronal slices (totaling a reconstruction with an anterior to posterior diameter of 180 mm) for each subject.

### Reader study/ data analysis

**Fig 2:**
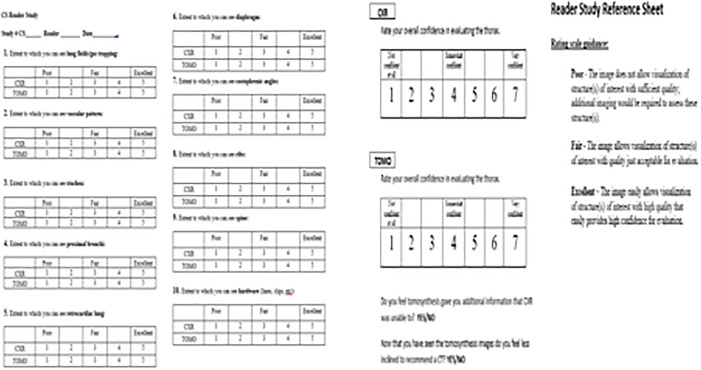
Reader score sheet provided to radiologist for both Chest X-ray and Stationary Digital Chest Tomosynthesis.

The s-DCT scans were compared to the portable CXR images by three board-certified thoracic radiologists (MS, BH, EP), all with at least 6 years of experience in interpreting clinical chest images. The reconstructed s-DCT image stacks were de-identified and were presented to the readers in a randomized order. Portable CXR scans and s-DCT scans were presented separately with a week wash-out period in between reads. Images were reviewed on an offline workstation with medical-grade 3-megapixel monitors. Standard image software evaluation tools (magnification, window/leveling) were available. Readers scored the quality of imaging on a scale of 1–5 based on ten criteria: visualization of lung fields, vascular pattern, trachea, proximal bronchi, retrocardiac lung, diaphragm, costophrenic angles, ribs, spine, and hardware (Fig 2). Readers were also asked to rate their confidence in interpreting the scans on a scale of 1–7. Furthermore, readers evaluated whether s-DCT offered them more information than CXR and whether a CT would be recommended for further evaluation.

### Statistical analysis

All statistical analyses were performed in SAS Version 9.4 (Cary, NC, USA). For each outcome, we evaluated whether there was a difference in mean outcomes by modality by implementing random effects models. Each outcome was separately modeled as a function of a modality indicator (s-DCT vs CXR). We assumed continuous errors for all outcomes except for ‘Hardware’, so that outcomes were modeled using a normal distribution with identify link. Hardware was dichotomized as ‘0’ and ‘greater than 0’ and modeled using a random effects logistic regression with a logit link.

For all models, we included the reader as a G-side random intercept and the patient as a compound-symmetric R-side random effect to account for correlation arising from the clustered nature of the data. A p-value for the parameter estimate of the modality indicator (<0.05) was considered evidence of a difference in the mean of the outcomes by modality. We included a point estimate for the difference in means for each outcome, the standard error for this difference, p-values for the test of the difference, and 95% confidence intervals for the difference.

We also evaluated the association between overall confidence and the following outcomes: whether the radiologist responded that s-DCT supplied additional information compared to plain film radiographs and whether a CT was recommended for further evaluation. The binary outcomes were modeled as a function of overall confidence. We included a G-side random intercept for the reader and an R-side compound-symmetric random effect for the patient to account for the correlated nature of the data. A p-value for the parameter estimate of the overall confidence (<0.05) was considered evidence of an association between overall confidence and the outcome. We provided predicted probabilities of the outcome at low (defined as the 10th percentile), median, and high (defined as the 95th percentile) values of overall confidence along with associated standard errors and 95% confidence intervals.

Each of the subjects successfully imaged were stratified into subcategories of disease processes based on radiologists’ impression of each subject’s s-DCT imaging. The subcategories used for disease processes reflected the wide variety of clinical pathology that could be imaged, and are as follows: infectious process (n = 3), lung nodules (n = 6), lung nodules with infectious process (n = 3), lung nodules and post-operative changes (n = 1), neoplastic process (n = 2), lung nodules and asbestosis (n = 1), post-op changes (n = 3), post-op changes and infectious processes (n = 2), and post radiation changes (n = 1).

## Results

A total of twenty-seven patients were recruited to the study. Technical failure resulted in 5 patients without usable imaging data. A total of twenty-two adult patients were successfully imaged (mean age, 65.6 years + /- 8.5; 15 women).

**Fig 3:**
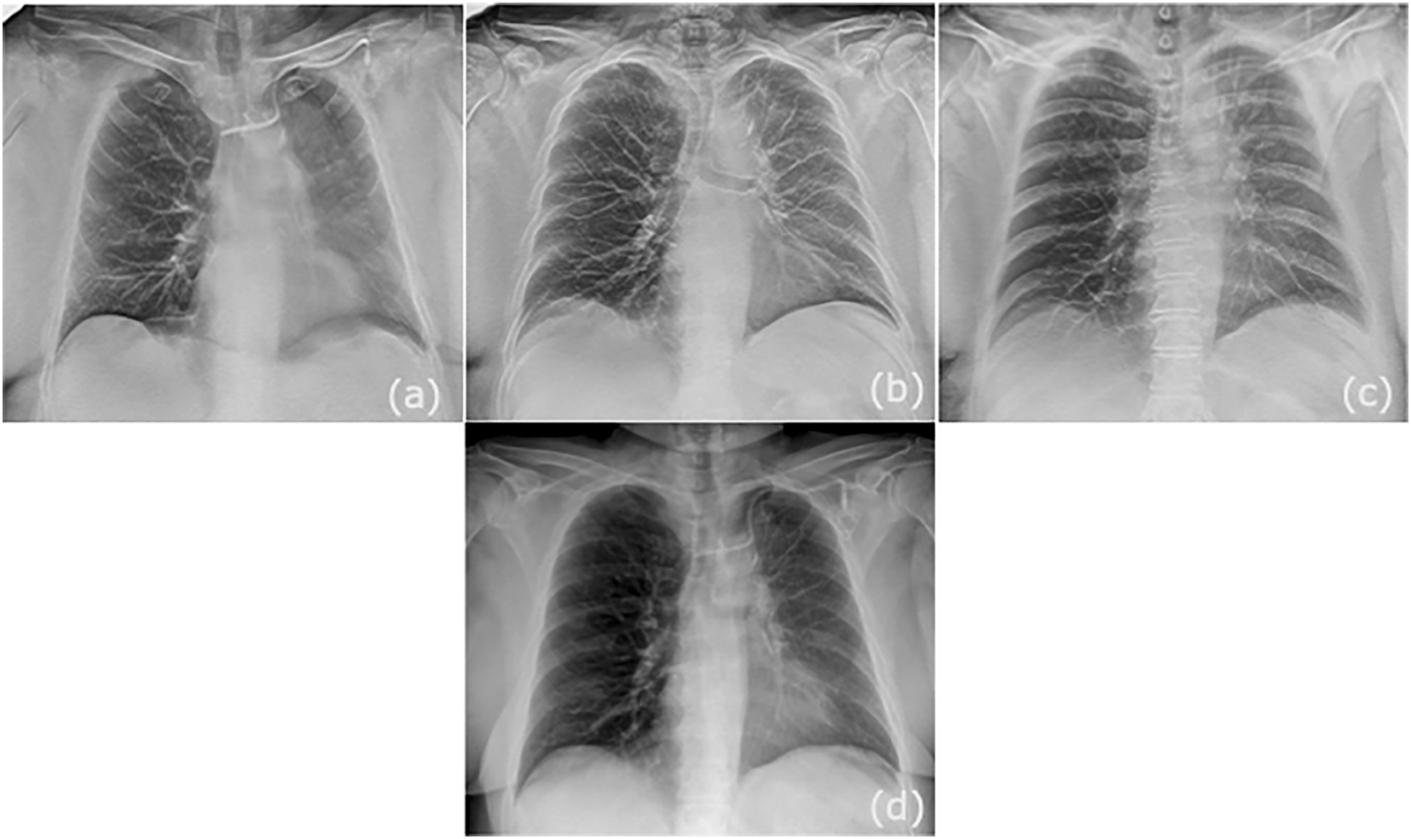
Representative coronal slices obtained on the s-DCT system (a-c) and corresponding CXR from the same patient (d). There is improved in-plane visibility of the left port-a-cath and line, proximal airways, and vasculature. The retrocardiac lung and osseous structures (ribs and spine) are better visualized in-plane given lack of superimposed structures.

**Fig 4:**
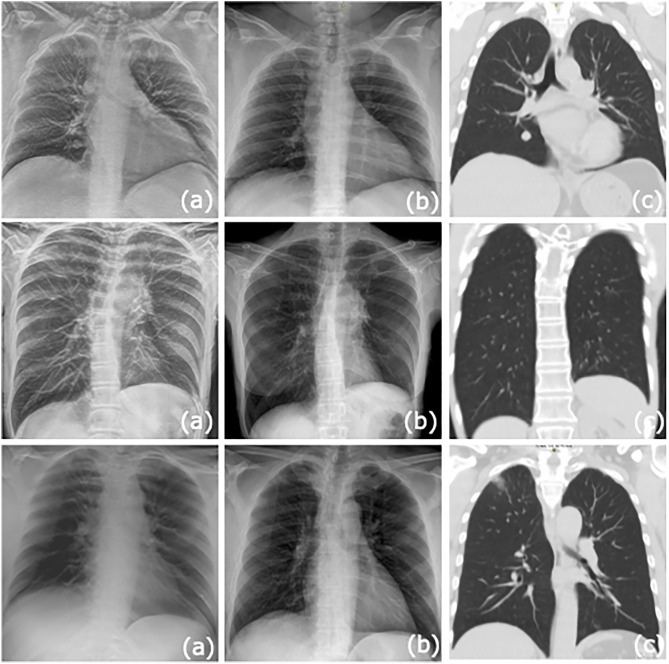
Comparison of single slices of s-DCT (a) with corresponding CXR (b). Comparison single coronal slice CT images are also provided (c). Row 1: Improved visualization of a 1.4 cm solid pulmonary nodule in the medial right middle lobe. The overlying right atrium limits evaluation on the CXR. Row 2: S-DCT and CXR show a dense left perihilar consolidation. Tomosynthesis demonstrates ground glass opacities in the retrocardiac left lower lobe, not seen on CXR, which are confirmed on chest CT. Row 3: S-DCT demonstrates a ground glass nodular opacity in the subpleural right upper lobe. Not well seen on CXR due to the overlying osseous structures. Findings are confirmed on chest CT.

By reducing overlap of anatomical structures with our s-DCT device, we were able to show improved visualization of previously visualized structures on CXR, as well as uncover completely new findings not seen on CXR (Figs 3 and 4).

Readers overall confidence with s-DCT was significantly higher than CXR (p = < 0.001). Examining the individual components, vascular pattern, proximal bronchi, retrocardiac lung, and osseous structures all had significantly higher confidence for s-DCT over CXR (Table 1). Additionally, the confidence in all other anatomic components also trended higher for s-DCT, except for the visualization of hardware which was equal between the two modalities.

**Table 1:** Difference in confidence of reader assessments of s-DCT versus CXR, by 10 separate criteria and overall, p-values and confidence limits. Positive calculated differences favor s-DCT. LUL = lower confidence limit, UCL = upper confidence limit.

Target criteria	Calculated Difference in Reader Confidence	Std Error	p value	LCL	UCL
Lung Fields/ Gas Trapping	0.1818	0.121	0.1344872	−0.057	0.421
Vascular Pattern	1.1061	0.106	<.0000001	0.896	1.316
Trachea	0.1818	0.126	0.152403	−0.068	0.432
Proximal Bronchi	1.2121	0.112	<.0000001	0.990	1.435
Retrocardiac Lung	0.4848	0.118	0.000066	0.252	0.717
Diaphragm	0.0152	0.118	0.8982742	−0.219	0.249
Costophrenic Angles	0.1970	0.121	0.1048741	−0.042	0.436
Ribs	0.8636	0.100	<.0000001	0.666	1.062
Spine	1.0606	0.107	<.0000001	0.849	1.272
Hardware	0.0000	0.045	1	−0.004	0.004
Overall Confidence	0.5758	0.165	0.0006534	0.250	0.902

We used the perception of the readers of whether additional information was provided by s-DCT and if they recommended a follow-up CT as additional metrics of confidence other than self-report. These metrics were not mutually exclusive. Self-reported overall confidence in tomosynthesis evaluations were grouped into 95% confidence intervals at low (10th percentile), median, and high (90th percentile). Higher overall confidence in tomosynthesis was associated with increased radiologists’ perception that tomosynthesis gave additional information that CXR did not (p = 0.05) (Table 2). A trend towards not recommending a follow up chest CT was noted with increased overall confidence in s-DCT, however this was not statistically significant (p = 0.47) (Table 2).

**Table 2:** Predicted probabilities of the outcomes 1) Reader perceived additional information was provided by s-DCT and 2) CT recommended as a follow up to s-DCT compared to low, median, and high confidence intervals of reader overall confidence in s-DCT evaluation.

Outcome	Percentiles of Confidence in s-DCT Evaluation	Predicted Probability	Std Error	LCL	UCL
Add. Information Provided	Low	0.37	0.28	0.052	0.86
Add. Information Provided	Median	0.55	0.26	0.13	0.91
Add. Information Provided	High	0.72	0.21	0.24	0.96
CT Recommended	Low	0.17	0.12	0.033	0.54
CT Recommended	Median	0.21	0.11	0.07	0.49
CT Recommended	High	0.27	0.12	0.097	0.55

**Fig 5:**
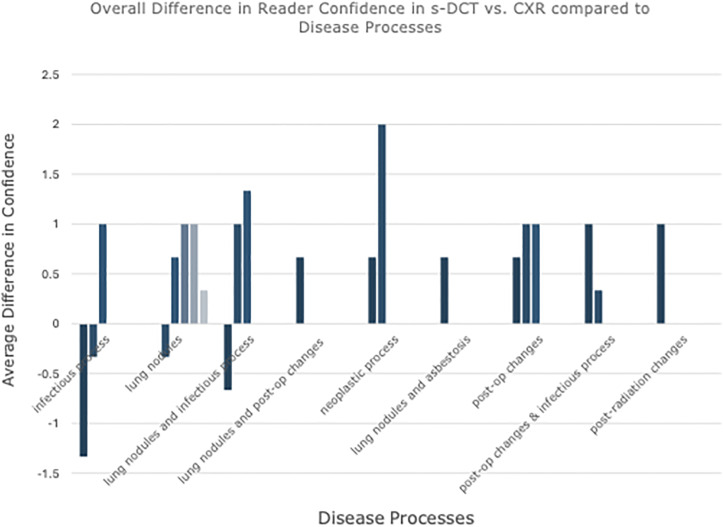
Average difference in overall reader confidence in s-DCT vs. CXR compared to disease processes. Each bar represents an average difference in confidence for each study, among three readers. A negative value favors confidence in CXR over s-DCT.

When successfully imaged subjects were split into categories based on disease processes, the mean value of the differences in overall reader confidence for each subject in s-DCT vs. CXR was positive, except for one category: infectious process (Fig 5). This suggests that readers had increased confidence in s-DCT over CXR in all categories except infectious processes.

It is expected that reader confidence in the visualization of some structures would be higher with s-DCT than CXR, given the modality’s superior capability to reduce overlapping anatomical structures. Notably, however, we found that s-DCT imaging uncovered findings completely missed by CXR. This suggests that implementing s-DCT in an ICU setting offers the potential for higher-sensitivity imaging. Additionally, higher reader confidence correlated with a lower predicted probability of CT recommendation, though without statistical significance. This offers the possibility of reduction in radiation exposure in populations requiring interval imaging in facilities opting for s-DCT over CXR. Interestingly, overall confidence in reader interpretation was lower for s-DCT than CXR in patients imaged with infectious processes, when averaged. In these patients, infectious processes (centrilobular groundglass nodules, pneumonia, and basilar opacities) were the main findings noted in the impressions. This may be due to radiologists’ greater familiarity and significantly more experience in diagnosing these processes on CXR vs. s-DCT, whereas post-op changes, neoplastic processes, post-radiation changes, and nodules are familiar findings in s-DCT on breast imaging. However, a clear reason is not yet understood. It is expected that radiologists should have increased confidence reading cases with multiple disease processes on s-DCT over CXR due in-plane visualization and lack of superimposition.

## Discussion

In the ICU, physicians depend on P-CXR because of the speed of acquisition and portability it provides in confirming correct placement of life-sustaining devices that are necessary to rapidly execute life-saving interventions. However, the low sensitivity, low contrast resolution, and lack of 3D detail of P-CXR images leaves much to be desired [26]. Notably, portable CXR has demonstrated low sensitivity in diagnosing common, urgent findings in the ICU, such as pneumothorax, pleural effusions, and pulmonary edema [4]. Although conventional CT is accepted as diagnostically superior to P-CXR in diagnosing thoracic pathologies common to the ICU, the lack of an accessible, portable chest CT system negates its diagnostic value for unstable ICU patients. With conventional CT, there remain risks and personnel requirements that complicate patient transport to the CT scanners in the radiology department [6,7]. Even in ICU patients that are stable enough for transport to CT imaging, the modality’s relatively high radiation dose, high cost, and limited access in high volume centers still prevent CT from being utilized for the monitoring needs of the ICU. Portable s-DCT overcomes these issues by remaining low cost, generating low radiation exposures, and providing the accessibility of a portable x-ray system. Conventional body imaging tomosynthesis systems are too large to be utilized as portable devices. Though conventional portable x-ray systems could offer a tomosynthesis acquisition, the mechanical movement of the unbalanced x-ray tube raises the potential for unstable gantries therefore leading to motion blurs in the tomosynthesis images. Utilizing a single tube with multiple x-ray emitters allows for a totally stationary acquisition platform and reduces motion blur associated with tube movement for projection image acquisition. Furthermore, CNT X-ray sources have the ability to perform gated imaging, which can be readily incorporated into a stationary system without the need for pausing tube motion. Performing respiratory gating could remove breath-hold requirements during imaging. [28,29] We have demonstrated that s-DCT imaging improves visualization of various thoracic structures compared to portable CXR. By improving resolution and reducing anatomy clutter of these structures, healthcare providers will be able to have a better understanding of disease progression or treatment response over time, thus opening the potential to adjust treatment plans more readily. Overall reader confidence in s-DCT was found to be higher than CXR, and as reader confidence in s-DCT increased so did the belief that tomosynthesis gives information that is not provided by CXR. This is clinically significant, as any additional information could vastly change patient management and improve patient outcomes.

The concept of a portable DCT system has been explored previously [9]. Cant et al. (2017) have performed simulated DCT using CT scans of ICU patients and a humanoid model and compared these findings to those with portable chest x-ray. Their findings revealed improved detection of pneumothorax, pneumomediastinum, and retrocardiac consolidations [27]. However, as with any system that relies on a moving x-ray source, the authors raise the issue of motion blur as a potential source of image quality degradation. Respiratory motion is also cited as a potential source of motion artifact, stemming from the inability of some ICU patients to hold their breath. Our stationary system avoids these issues given the lack of x-ray source motion and the potential for rapid image acquisition speed. Additionally, previous work has demonstrated the ability to perform prospective physiologic– both respiratory and cardiac gating with the s-DCT system, which could be further utilized in the ICU setting to reduce motion artifact [28,29].

We have demonstrated that our system is capable of providing clinically impactful information in the management of the general outpatient population, with future directions to study specific patient inpatient populations. Furthermore, our study illustrates the potential for the use of an s-DCT system in bedside image guided procedures where multi-plane imaging would be useful for accurate guidance and for reduction in procedure times [18]. Other groups have evaluated the use of tomosynthesis for looking at interstitial lung disease compared to conventional radiography, showing statistically significant improvements in diagnostic confidence, interobserver agreement, and sensitivity [30]. The imaging findings of our study were consistent with those of prior studies, namely that proximal bronchi, small airways and pulmonary vasculature have improved visualization on s-DCT compared with chest x-ray [9].

In the evaluation of patient hardware, our study did not utilize either the respiratory or cardiac gating capability of the CNT based system, which likely led to increased motion blur of hardware during the 5.8 seconds of 29 projection image acquisition. This likely led to the lack of difference between the s-DCT and CXR scores. Recent studies using conventional thoracic tomosynthesis have shown improved sensitivity and accuracy detecting silicone airway stents and complications over CXR [31], confirming the capabilities of digital tomosynthesis over CXR. We intend to examine the respiratory and cardiac gating capability of our system for the evaluation of hardware and for evaluating small hyperdense structures such as coronary artery calcium in the future.

Some limitations are evident in our study. Our study was limited to 27 outpatients. Our goal was to explore a potential mobile tomosynthesis setup in a small patient population for both feasibility and to understand potential clinical utility. Our study population included patients receiving outpatient CTs for pulmonary conditions rather than patients in the ICU. At this stage in development the s-DCT system was not located in the ICU, and therefore we were unable to image this patient population. Our prototype system utilized a repurposed x-ray tube designed for airport baggage security inspection that is limited to 80kVp, lower than the more typical 100–120 kVp used for chest tomosynthesis and radiography, which may result in lower penetration of more radio-dense structures. One other limitation of this study was the limited angular coverage of this specific x-ray source; the use of a longer X-ray tube could increase the angular coverage. Conventional chest tomosynthesis imaging typically spans 30–45 degrees, but our repurposed tube provided only 12 degrees at our source-detector distance, with a resultant increase in our artifact spread function. It is important to note that the longer tube length required to produce a wider, conventional angular range would be prohibitive for this project, which requires a tube length that can move in and out a portable application. However, neither the source kVp or source length used in this study are fundamental limitations of the CNT based x-ray sources, for example, our team has demonstrated the use of higher energy sources for stationary head CT imaging [32,33]. Furthermore, we have a longer one-meter tube under evaluation for chest imaging and intend to explore the tradeoff between tube length and lesion conspicuity. Future research using s-DCT in the ICU could potentially provide further information towards the benefits of tomosynthesis. There was also some variation in reader scoring and confidence, this can be attributed to reader’s unfamiliarity with tomosynthesis scans. Finally, though there was a trend towards not recommending a follow up chest CT based on the tomosynthesis images, this was not statistically significant. Further study with more critically ill patients would help inform the clinical utility of such a system.

In conclusion, stationary digital chest tomosynthesis is a superior alternative to portable chest x-ray for patients in the ICU setting that cannot undergo CT examination. Further work needs to be done to more fully assess s-DCTs utility in the ICU and how its use could reduce the need for CT examinations.

## Supporting information

S1 File Supporting Data. (XLSX)
